# Post-cycle therapy after androgen abuse: a narrative review of mechanisms, evidence, and clinical perspectives

**DOI:** 10.1530/EC-26-0319

**Published:** 2026-09-10

**Authors:** Diederik L Smit, Tijs Verdegaal, Peter Bond, Willem de Ronde

**Affiliations:** ^1^ Department of Internal Medicine, Zaandam Medical Centre, Zaandam, the Netherlands; ^2^ Android Health Clinic, Department of Performance and Image-enhancing Drugs Research, Utrecht, the Netherlands; ^3^ Department of Internal Medicine, Spaarne Gasthuis, Haarlem, the Netherlands; ^4^ Department of Endocrinology and Metabolism, Amsterdam UMC, Amsterdam, the Netherlands

**Keywords:** anabolic steroids, androgen abuse, bodybuilding, testosterone, hypogonadism, post-cycle therapy, harm reduction

## Abstract

Post-cycle therapy (PCT) refers to the use of pharmacological agents – most commonly selective estrogen receptor modulators (SERMs), human chorionic gonadotropin (hCG), and aromatase inhibitors (AIs) – after cessation of androgen abuse to accelerate recovery of the hypothalamic–pituitary–gonadal (HPG) axis. Exogenous androgens suppress endogenous testosterone production through negative feedback, often resulting in a transient hypogonadal state that may persist for weeks to months, and occasionally longer, depending on cycle characteristics and individual factors. The rationale of PCT is to mitigate this suppression and reduce symptoms of androgen deficiency. However, evidence supporting its use is limited, with most data derived from observational studies or extrapolated from other forms of secondary hypogonadism. While some studies suggest that PCT may accelerate short-term hormonal and seminal recovery, spontaneous recovery occurs in the majority of individuals within months after cessation. Three perspectives can be distinguished. In real-world practice, PCT is widely used, often without medical supervision, driven by cultural norms and concerns about symptom persistence. From an academic standpoint, PCT is generally not recommended because of uncertain benefits and potential risks. A third perspective describes a pragmatic rationale for considering a short course of PCT in carefully selected individuals with significant symptom burden or risk of relapse into androgen abuse, while acknowledging that this approach is not supported by robust clinical evidence. This review aims to clarify the rationale, evidence gaps, and clinical implications of these perspectives to support informed clinical decision-making.

## Introduction

Anabolic-androgenic steroids (AAS) – hereafter referred to simply as androgens – are a common form of performance- and image-enhancing drugs (PIEDs) that are used among both competitive and recreational athletes. Androgen abuse poses a public health concern as its prevalence rises (1, 2), and it clearly carries health risks, including cardiovascular disease and heart failure (3, 4, 5), infertility and impaired spermatogenesis (6, 7), psychiatric disturbances (8, 9), and in some individuals prolonged or potentially irreversible suppression of endogenous testosterone production (10, 11).

Traditionally, androgens are taken in cycles, with periods of supraphysiological exposure followed by complete cessation (12). The hypogonadal state after discontinuation can persist for weeks to months and may involve fatigue, low libido, depressed mood, and loss of muscle mass (7, 13, 14). These symptoms are a major driver for initiating post-cycle therapy (PCT), which has therefore become a widely adopted strategy aimed at facilitating recovery of the hypothalamic–pituitary–gonadal (HPG) axis. PCT typically involves selective estrogen receptor modulators (SERMs), human chorionic gonadotropin (hCG), and/or aromatase inhibitors (AIs), with the intended goal of accelerating the restoration of endogenous testosterone production and reducing symptoms of androgen deficiency (15, 16). Although firmly embedded in the culture of androgen abuse, PCT is not part of any formal medical guideline and remains largely unsupported by controlled clinical evidence. In addition, many individuals experience symptoms during androgen withdrawal, despite PCT, or fear them to such an extent that they adopt a ‘blast-and-cruise’ approach, meaning they do not cease androgen abuse at all and alternate cycles with a lower, albeit usually still supraphysiological, maintenance dose. This practice increases cumulative androgen exposure and the risk of long-term health consequences, including permanent endocrine damage and premature atherosclerosis (17, 18).

Clinicians from different disciplines – including endocrinology, general practice, sports, and addiction medicine – are increasingly confronted with patients recovering from androgen abuse. Yet, guidance on how to manage post-cycle recovery is scarce, and perspectives on PCT vary widely between evidence-based medicine, pragmatic harm-reduction approaches, and user-driven practice. This review is written for this broad clinical audience, aiming to clarify these contrasting paradigms rather than to advocate any single strategy. Given the limited, heterogeneous, and predominantly observational nature of the available literature, this article was intentionally designed as a narrative review to explore and contextualize these differing perspectives rather than to provide a formal systematic evidence synthesis. By critically examining the mechanistic rationale, available evidence, and clinical viewpoints on PCT, this review seeks to bridge the gap between real-world practice and scientific understanding and to provide a framework for informed dialog with individuals recovering from androgen abuse.

### Mechanistic background of hypogonadism after androgen abuse

**Figure 1:**
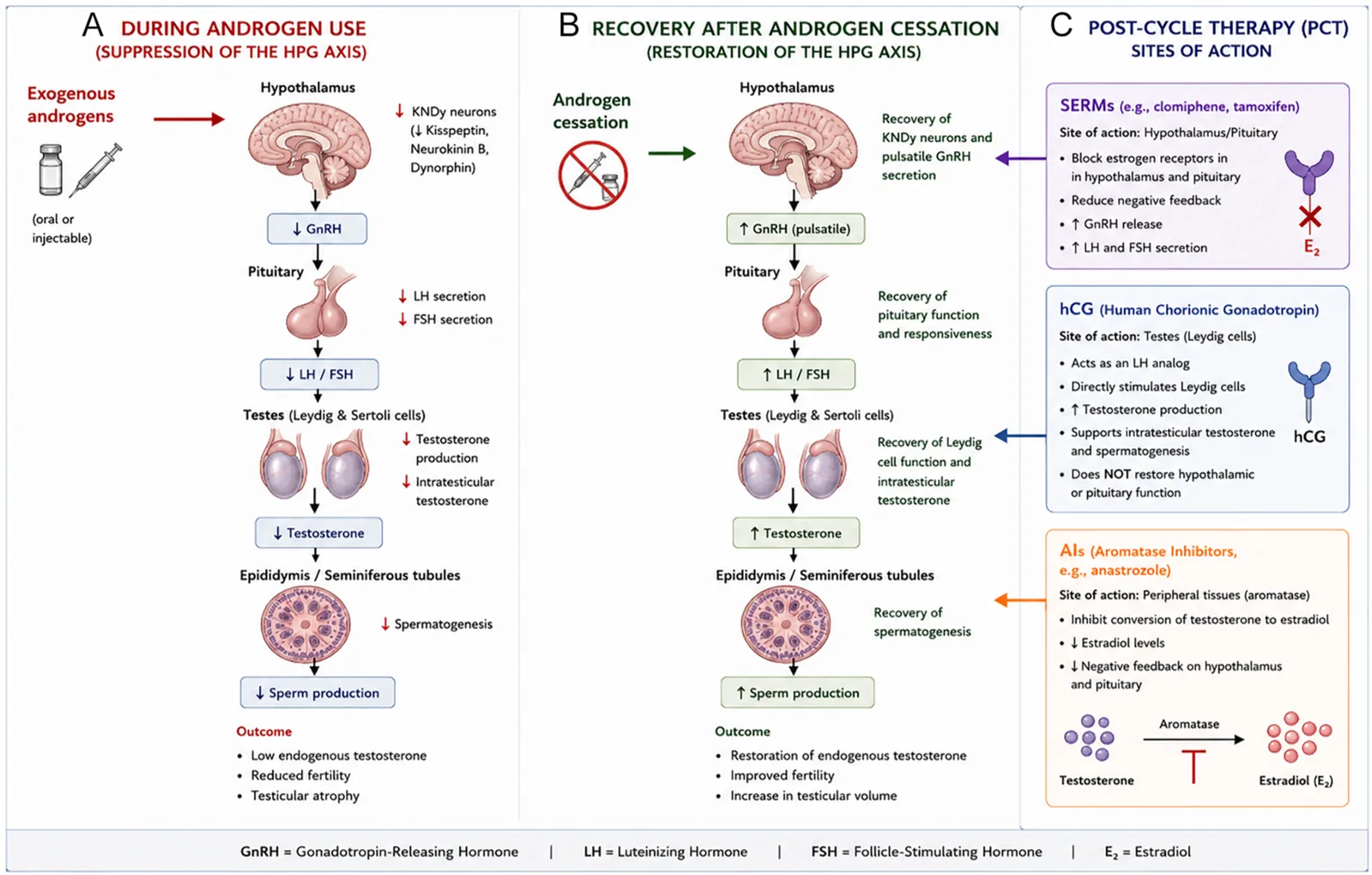
Mechanisms of HPG axis suppression and recovery after androgen abuse and proposed sites of action of post-cycle therapy agents.

Exogenous androgens exert profound suppressive effects on the HPG axis through negative feedback mechanisms at both the hypothalamic and pituitary levels (19). Normally, pulsatile secretion of gonadotropin-releasing hormone (GnRH) from the hypothalamus drives the release of luteinizing hormone (LH) and follicle-stimulating hormone (FSH) from the anterior pituitary, which in turn stimulate testicular Leydig and Sertoli cells to produce testosterone and support spermatogenesis, respectively. When supraphysiological concentrations of androgens are introduced exogenously, circulating androgen levels rise far beyond the physiological range. Moreover, a substantial fraction of these androgens undergoes aromatization to estrogenic metabolites in peripheral tissues, such as adipose tissue, leading to high serum estrogen levels. Direct feedback inhibition at the hypothalamic level can be mediated by both androgens and estrogens, whereas at the pituitary level, suppression is thought to depend predominantly on aromatization to estrogens, since gonadotrophs express estrogen receptors but little to no androgen receptors (20). Accordingly, estrogenic metabolites act primarily at the pituitary, while both androgens and estrogens influence hypothalamic neurons, together reducing GnRH release and blunting pituitary responsiveness to GnRH stimulation (19, 21). GnRH neurons themselves lack sex steroid receptors, and feedback inhibition by sex steroids is therefore thought to be mediated by intermediary neuronal populations (22). The most likely candidates are the KNDy neurons – named for their co-expression of kisspeptin, neurokinin B, and dynorphin – located in the infundibular nucleus (the human homolog of the arcuate nucleus in many other animal species). These neurons constitute the intrinsic GnRH pulse generator, which governs the frequency and amplitude of GnRH release (22, 23). Estradiol, in particular, strongly modulates these neurons via estrogen receptor *α* (24), thereby slowing the pulsatile signaling that drives GnRH release and dampening GnRH responsiveness at the pituitary (25). Testosterone and dihydrotestosterone can also modulate hypothalamic GnRH secretion and pituitary gonadotropin release through androgen receptor signaling. The combined effect is an abrogation of LH and FSH secretion, leading to loss of endogenous testosterone production, impaired spermatogenesis, and testicular atrophy (see Fig. 1) (13).

When exogenous androgens are discontinued and circulating concentrations decline, the HPG axis does not immediately resume its physiological activity, for reasons that are incompletely understood. The period of prior suppression alters hypothalamic GnRH pulse generation and pituitary sensitivity, such that both GnRH secretion and the responsiveness of gonadotrophs to GnRH stimulation remain blunted for some time (11, 26). Clinical and observational studies suggest that the duration of HPG axis suppression is broadly proportional to the duration and intensity of androgen exposure, with longer or higher-dose regimens leading to more protracted recovery periods (7, 10, 12, 27). Delayed testosterone recovery has also been reported in men undergoing androgen deprivation therapy for prostate cancer, where suppression is induced centrally through long-acting GnRH agonists rather than by peripheral androgen excess; nonetheless, the clinical pattern of slow and sometimes incomplete HPG axis recovery is remarkably similar (28, 29). A comparable association between treatment duration and recovery time has been demonstrated in male hormonal-contraceptive trials, where longer exposure delays the return of spermatogenesis (30). Slow normalization of gonadotropins, up to 12–15 months, has likewise been noted after discontinuation of long-acting testosterone undecanoate, although this mainly reflects the extended half-life of the depot and concomitant progestagen use (31). It is during the post-cycle period that men therefore often experience symptoms of hypogonadism such as fatigue, depressed mood, sexual dysfunction, or loss of muscle mass, but the severity of these symptoms varies considerably between individuals (6, 32). Factors such as age, baseline gonadal function, and psychological tolerance for hypogonadal symptoms appear to modulate both the perception and impact of this withdrawal state, with older men and men with lower baseline testosterone demonstrating slower or less complete recovery (13, 14).

### Principle of PCT

The central question in recovery after androgen abuse is where the main bottleneck in HPG axis recovery resides. Both central and testicular dysfunctions are observed: androgens and their estrogenic metabolites impair KNDy neuron activity, GnRH secretion, and pituitary sensitivity to GnRH, while simultaneously inducing testicular atrophy and impaired spermatogenesis. Stimulation testing has demonstrated blunted LH responses to GnRH and reduced testosterone responses to hCG, indicating impairment at both central and testicular levels (33, 34). Clinical cohorts, however, show that men who remain hypogonadal after cessation most often present with inappropriately low LH and FSH, consistent with a central hypogonadism phenotype (10, 11, 13). Thus, while evidence is somewhat conflicting, the prevailing clinical picture points to hypothalamic–pituitary suppression as the main limiting step, with a contributory but usually less dominant testicular component, suggesting that testicular dysfunction may be less persistent than central suppression in many individuals.

The rationale of PCT is to intervene pharmacologically during this vulnerable period in an attempt to prevent, or at least shorten, the duration of hypogonadism. In practice, men often begin PCT once exogenous androgens start to clear from the circulation, timing the initiation according to the half-life of the compounds used – earlier after oral or short-acting agents, later after long-acting esters (14, 16, 27). The most frequently used drugs are SERMs, such as clomiphene citrate or tamoxifen, which act centrally by blocking estrogenic negative feedback inhibition at the pituitary and hypothalamus, thereby stimulating GnRH and subsequent LH and FSH release. Some protocols incorporate hCG to directly stimulate Leydig cells in the testes to produce testosterone. However, hCG does not support recovery of the hypothalamic–pituitary axis, as it stimulates testicular testosterone production, which in turn suppresses LH and FSH secretion through negative feedback, with higher doses leading to more profound gonadotropin suppression (35). While it can temporarily normalize testosterone production, this effect does not reflect restoration of axis function and may give a misleading impression of recovery. Consequently, normalization of serum testosterone concentrations should not automatically be interpreted as evidence of recovery of physiological HPG axis function. Aromatase inhibitors are occasionally added to suppress estradiol concentrations and reduce negative feedback. PCT regimens are typically continued for several weeks to months, with intended outcomes of normalizing testosterone and gonadotropin concentrations, relief of hypogonadal symptoms, and preservation of muscle mass and physical performance (7, 12).

### The real-world landscape of PCT

To understand the contrasting perspectives on PCT, it is useful to first consider how the practice unfolds in the real world. Although the term PCT is widely used by individuals who abuse androgens, clinicians, and researchers, it does not refer to a single standardized intervention. Rather, PCT encompasses a heterogeneous collection of practices that vary considerably in composition, timing, duration, dosing, and intended outcomes. Among individuals who abuse androgens, PCT is highly prevalent, although reported rates vary across studies. In a large retrospective single-center survey, 56.5% reported using PCT after androgen discontinuation (36), whereas a prospective Dutch cohort study found that nearly 80% initiated PCT during follow-up (16). Together, these data illustrate that PCT is perceived as an important step, sometimes even after very short cycles when a quick spontaneous recovery would be expected. Within bodybuilding communities, PCT is frequently portrayed as indispensable, with online discussions and coaches often warning that recovery is severely impaired without pharmacological support.

The PCT regimens adopted are characterized by considerable heterogeneity, with protocols circulating online or through peer networks rather than through medical channels. The choice of compounds, dosing, timing with respect to the last androgen dose, and duration often resembles a ‘wild west’ of self-experimentation, compounded by the fact that most agents are obtained from underground laboratories of uncertain quality. A notable example is the widespread inclusion of hCG in PCT regimens: while high-dose use is popular among users, its administration may counteract the intended purpose of facilitating endogenous recovery because it can reduce pituitary gonadotropin release. Another frequently reported practice is the concurrent use of two SERMs, typically tamoxifen and clomiphene, in the belief that combining them provides additive stimulation of the axis. Some individuals include mesterolone (Proviron) in their PCT protocols, despite the fact that it is itself an androgen and may actually contribute to further suppression (16). Furthermore, the wide range of symptoms during the post-cycle ‘dip’ makes it difficult to disentangle symptoms related to androgen withdrawal from those caused by the PCT regimen. Moreover, the fact that the vast majority of men ultimately recover after an androgen cycle (13) can easily create the impression that recovery occurred *because of* the PCT rather than in spite of it. Together, these dynamics reinforce the perception of PCT as both effective and safe.

### The academic perspective on PCT

From an academic point of view, the effectiveness of PCT after androgen abuse remains highly questionable. Evidence from randomized controlled trials is extremely limited. A recently conducted placebo-controlled trial investigating letrozole in former androgen users with low testosterone was terminated prematurely because of poor recruitment and was therefore unable to provide definitive conclusions regarding efficacy (37). Consequently, the available evidence remains largely restricted to observational studies and self-reported outcomes. A few expert reviews have proposed potential approaches, including the use of SERMs, hCG, or AIs (17, 38, 39), but these recommendations are based on theoretical reasoning and clinical experience rather than controlled data, and none are endorsed in formal guidelines. In a survey among athletes who abuse androgens, many reported subjective benefits such as reduced withdrawal symptoms and mood disturbances (40). A retrospective analysis found that men who performed PCT achieved normalization of reproductive hormones in a median of 13 weeks, compared with 26 weeks in those who did not, with the apparent advantage being limited to the first 3 months after discontinuation and disappearing thereafter (36). In the prospective HAARLEM cohort, no difference in endogenous testosterone concentrations at 3 months was observed between participants who elected to use PCT and those who did not (13). However, the study was not designed to evaluate PCT efficacy, and most participants were recovering from a single androgen cycle, limiting causal inference and generalizability to individuals with more prolonged androgen exposure.

One of the more informative datasets comes from a recent retrospective cohort study in recreational athletes who abuse androgens with relatively limited exposure, in whom outcomes after expectant management were compared with clomiphene monotherapy or clomiphene combined with hCG (41). In this study, pharmacological PCT was associated with a more rapid recovery of endogenous testosterone levels, sexual function, and semen parameters – although this should not necessarily be interpreted as restoration of physiological HPG axis function – compared with no treatment, with the most pronounced effects observed in the combined group. Importantly, however, hormonal recovery occurred across all groups over time, with near-normalization by 6 months, indicating that spontaneous recovery remains the rule rather than the exception. These findings raise the possibility that PCT may accelerate some aspects of short-term recovery, but do not establish a clear long-term benefit. Moreover, the study is limited by its retrospective design, the inclusion of a highly selected population with relatively short androgen exposure and normal pre-cycle reproductive function, and variability in treatment duration and adjunctive therapies.

An additional challenge is that biochemical recovery and symptomatic recovery should not be considered synonymous. While several studies have focused on serum testosterone concentrations as a primary outcome, the relationship between hormonal recovery and symptom improvement appears imperfect. Persistent symptoms following androgen cessation may occur despite normalization of testosterone concentrations, whereas some individuals report symptomatic improvement in the absence of substantial biochemical changes (42). This observation suggests that biochemical hypogonadism may not fully explain the post-cycle experience in all individuals. Psychological factors, expectations regarding recovery, and the effects of withdrawal from prolonged supraphysiological androgen exposure may all contribute, although their relative importance remains poorly understood (40, 43). Consequently, improvements in laboratory parameters should not automatically be interpreted as evidence of meaningful clinical benefit.

Restoration of fertility deserves separate consideration from recovery of endogenous testosterone production. While many users pursue PCT to alleviate hypogonadal symptoms, others hope to accelerate recovery of spermatogenesis. These outcomes should not be considered interchangeable, as normalization of serum testosterone does not necessarily indicate restoration of fertility. Some observational studies have reported improvements in semen parameters among men receiving pharmacological interventions after androgen cessation, particularly when gonadotropin stimulation is enhanced through agents such as hCG or SERMs (41, 44). However, spontaneous recovery of spermatogenesis occurs in the majority of men after androgen discontinuation (6, 7, 13). Recovery may nevertheless take many months and appears to be influenced by factors such as age, cumulative androgen exposure, and baseline reproductive function (6, 7). At present, robust controlled data demonstrating that PCT improves long-term fertility outcomes remain lacking.

The pharmacological agents commonly employed in PCT are themselves not without risks. Adverse events related to SERMs, AIs, and hCG have been reported, ranging from sexual dysfunction, mood disturbances, and joint pain to retinal, hepatic, and cardiovascular complications (45, 46). Importantly, some of these adverse effects may overlap with the symptoms that PCT is intended to alleviate, complicating clinical assessment. Aromatase inhibitors may excessively suppress estradiol concentrations, potentially affecting libido, sexual function, bone metabolism, and general well-being, whereas SERMs have been associated with visual disturbances, mood changes, and thromboembolic events in rare cases (46, 47). These considerations further complicate the risk–benefit assessment of PCT, particularly given the limited evidence supporting its efficacy. A further challenge is that most PCT drugs are sourced from underground laboratories. A recent chemical analysis showed that over one-third of tested products were mislabeled or lacked the declared substance (45). This hampers any reliable evaluation of PCT effectiveness in real-world settings and highlights the need for controlled trials using pharmaceutical-grade compounds. One of the few prospective interventional studies in this field is the Norwegian CLoTASH pilot study, in which men with hypogonadism following long-term androgen use were treated with clomiphene citrate, with optional adjunctive hCG. The study reported improvements in testosterone concentrations and symptoms, but its interpretation is limited by the lack of randomization, absence of a placebo control group, small sample size, and the optional use of hCG (48). The interpretation of the study is further complicated by the optional use of testosterone or hCG, as both agents may counteract endogenous reactivation of the HPG axis, increasing the likelihood of a negative outcome in this study.

Taken together, the available literature does not support a clear benefit of PCT on long-term recovery of the HPG axis or hypogonadal symptoms, although limited observational data suggest a possible acceleration of short-term recovery. Importantly, most studies assess serum testosterone concentrations rather than sustained recovery of physiological HPG axis function after discontinuation of therapy, making interpretation of long-term endocrine recovery challenging. Instead, the most rational approach from an academic perspective is expectant management: reassuring patients that most men recover spontaneously and monitoring endocrine recovery with periodic blood tests, rather than initiating pharmacological intervention.

### The pragmatic case for PCT

Within the medical community, views on PCT vary considerably. While most clinicians would advocate expectant recovery, a pragmatic perspective has also been described, particularly in settings where clinicians seek to reconcile patient expectations with limited evidence and real-world constraints. Indeed, survey data suggest that a minority of endocrinologists prescribe pharmacological agents in this setting (49). The aim here is not to endorse such approaches, but to understand their rationale. While the scientific evidence is limited and methodologically weak, observational data suggest potential short-term benefits. Some of the aforementioned studies have reported shorter time to hormonal recovery and lower rates of withdrawal symptoms, depressive mood, and relapse into androgen abuse among men who initiated PCT (36, 40, 41). Although these findings are subject to selection bias, confounding, and expectancy effects, they indicate that, for some individuals, PCT may confer subjective or short-term improvements. These observations have contributed to a line of reasoning in which some have argued that a therapeutic trial might be considered, balancing limited and uncertain evidence against potential short-term benefits in selected individuals. This approach resembles other areas of endocrinology where treatment is occasionally considered in selected symptomatic individuals despite limited or conflicting evidence, such as the use of liothyronine (T3) alongside levothyroxine (T4) in hypothyroidism with persistent symptoms (50).

Beyond its potential physiological effects, psychological and behavioral factors play an important role in the post-cycle period. Many individuals who abuse androgens report concerns about persistent hypogonadism, loss of muscle mass, and mood disturbances after discontinuation. These concerns may influence both symptom perception and decision-making and are frequently cited as reasons for initiating PCT. In addition, placebo and expectancy effects may contribute to perceived benefits of PCT. Within bodybuilding and fitness communities, PCT is widely regarded as an essential component of responsible androgen use, and many users expect it to facilitate recovery and prevent adverse outcomes. Such expectations may influence symptom perception, treatment satisfaction, and confidence in the recovery process, independent of any direct pharmacological effect. Furthermore, the act of initiating a structured intervention may provide reassurance and reinforce a sense of control during a period often characterized by uncertainty regarding hormonal recovery. These factors should be considered when interpreting self-reported benefits of PCT, particularly in the absence of placebo-controlled trials. Indeed, surveys indicate that users commonly perceive PCT as a harm-reduction strategy and prefer to undertake it under medical supervision (40, 51). At the same time, perceived reassurance or expectation of benefit alone should not be considered a sufficient justification for pharmacological intervention. While acknowledging these concerns is an important part of clinical care, treatment decisions should not be driven by the desire to offer an active intervention in the absence of clear evidence.

Within this broader clinical context, one proposed perspective is that PCT may be viewed within a harm-reduction framework in carefully selected cases as a time-limited intervention, using low doses, frequent laboratory monitoring, and explicit risk–benefit evaluation. This perspective is intended to describe a pragmatic clinical rationale rather than an evidence-based recommendation. According to this perspective, such cases may include individuals with a high burden of hypogonadal symptoms or those at increased risk of relapse into androgen use due to difficulty tolerating the post-cycle state. Within this framework, this approach would generally be reserved for individuals with the intention to discontinue androgen abuse and is unlikely to be appropriate in those who plan to resume androgen abuse in the short term. If such an approach is adopted, it should always be accompanied by explicit informed consent and clear communication about the uncertainty of efficacy and possible side effects. Patients must be informed that most men recover spontaneously, that the effectiveness of PCT itself remains unproven, and that no formal guidelines currently recommend its use, although some expert commentaries have nevertheless described PCT as a potential strategy in the clinical management of patients who abuse androgens (17, 38, 39).

### Integrating practice, evidence, and pragmatism

The debate around PCT illustrates the tension between real-world practice, scientific evidence, and pragmatic harm-reduction strategies. In the real world, PCT is embedded as a near-obligatory ritual, sustained by cultural norms and psychological reassurance for men abusing androgens who fear hypogonadism after cessation. From an academic perspective, however, there is no convincing evidence that PCT supports recovery of the HPG axis, although limited observational data suggest a possible acceleration of short-term recovery. In the meantime, the risks of pharmacological agents themselves and the unreliability of underground products argue against its use. The pragmatic view occupies a middle ground, acknowledging that, while the intervention is unproven, it may be considered in carefully selected cases with significant symptom burden or risk of relapse, particularly in individuals with the intention to permanently discontinue androgen abuse. These perspectives are not mutually exclusive but represent different layers of the same reality: PCT is simultaneously a cultural practice, a scientific question, and a clinical dilemma.

Beyond its pharmacological rationale, PCT functions as a cultural and psychological construct within bodybuilding and fitness communities. For many users, it symbolizes control and recovery, regardless of a demonstrable biological benefit. This disconnect between community practice and academic evaluation widens the gap between patients and professionals. Categorical rejection risks alienating men who abuse androgens, whereas cautious engagement can foster dialog and trust. The broader lesson is that doping medicine cannot rely solely on evidence-based conservatism; it must also navigate the realities of user behavior, expectations, and anxieties. Pragmatic clinical approaches, even if imperfect, may therefore provide a bridge between scientific skepticism and lived experience. Ultimately, only well-designed studies can clarify whether PCT represents more than a cultural practice and provide an evidence-based foundation for clinical decision-making.

### Future directions for research

Conducting a randomized, double-blind trial with pharmaceutical-grade compounds is essential to determine whether PCT provides benefits beyond reassurance and spontaneous recovery. Such a study should include standardized symptom questionnaires, quality-of-life measures, and frequent endocrine assessments – ideally on a weekly basis – to capture subtle, short-term effects that may be missed at longer follow-up intervals. Particular attention must be paid to confounders, including cumulative duration of past androgen abuse, types of compounds used, and pharmacokinetics – particularly the half-life of the last androgen esters administered – because these factors strongly influence recovery dynamics. To minimize heterogeneity, a clearly defined protocol is needed, with clomiphene citrate as the most plausible candidate, given its potency, relatively extensive study base, and demonstrated short-term safety in men (52, 53). The optimal timing of initiation, dosing regimen, and treatment duration should be prospectively evaluated within such trials rather than inferred from existing evidence or clinical practice. By disentangling biological efficacy from psychological reassurance, such research could finally establish whether PCT has a measurable role in facilitating recovery after androgen abuse.

## Conclusion

PCT remains a contested practice, positioned at the intersection of culture, psychology, and clinical endocrinology. The aim of this review has been not to advocate for or against PCT but to highlight the diverse perspectives that shape its use and the tensions between them. For some, PCT represents an indispensable ritual; for others, an unproven intervention best avoided; and for yet others, a pragmatic harm-reduction tool. These divergent views underscore that clinical decision-making in this area remains complex and value-laden. The most appropriate approach will depend on clinical context, the available evidence, and the needs and expectations of the individual patient.

## Declaration of interest

The authors declare that there is no conflict of interest that could be perceived as prejudicing the impartiality of the work reported.

## Funding

This work did not receive specific project-based funding. The authors’ research group was supported by an unrestricted grant from the Dutch Anti-Doping Authority.

## Author contribution statement

DLS was involved in drafting and writing the manuscript. TV, PB, and WdR were involved in reviewing the manuscript.

## Ethics approval

No ethical approval was required for this study as this is a narrative review based exclusively on previously published literature and did not involve the collection or analysis of original human participant data.
